# Delayed bleeding after peroral endoscopic myotomy for achalasia: conquered by clearing tunnel clots and identifying the bleeding site

**DOI:** 10.1055/a-2688-6404

**Published:** 2025-09-18

**Authors:** Jiayao Zheng, Shijie Yang, Wanyin Deng

**Affiliations:** 1 117861 Department of Digestive Endoscopy Center, Fujian Provincial Hospital, Fuzhou, China; 2 117861 Fuzhou University Affiliated Provincial Hospital, Fuzhou, China; 3 Shengli Clinical Medical College of Fujian Medical University, Fuzhou, China

A 35-year-old man presented with dysphagia accompanied by regurgitation for 2 years, with his symptoms having worsened over the previous 3 months. After a series of comprehensive examinations, he was diagnosed with type 2 achalasia and underwent a successful peroral endoscopic myotomy (POEM). He developed chest pain 9 hours after the operation, which was accompanied by a small amount of hematemesis, and persisted without relief. An emergency gastroscopy indicated bleeding within the tunnel, so endoscopic hemostasis was performed with the patient under general anesthesia with tracheal intubation.

During the gastroscopy, the tunnel was seen to be obviously swollen, and bleeding within the tunnel was therefore considered. The tunnel opening was re-established endoscopically, and a large number of dark red blood clots were observed ( Fig. 1 **a** ). Removing the blood clots from the tunnel and finding the bleeding site were the key and most challenging aspects in achieving hemostasis. At first, a snare was tried to clear the blood clots; however, the tunnel filled with blood clots lacked sufficient space to open the snare. In contrast, with a clip, it was possible to more effectively grasp and remove the blood clots with fibrosis, thereby improving the efficiency of blood clot clearance. After spending 50 minutes clearing all the blood clots from the tunnel ( Fig. 1 **b** ), we identified active oozing of blood from a blood vessel in the submucosa at the cardia ( Fig. 1 **c** ). Hemostasis was performed by electrocoagulation under endoscopic visualization and was successfully achieved ( Fig. 1 **d** ; Video 1 ).

**Fig. 1:**
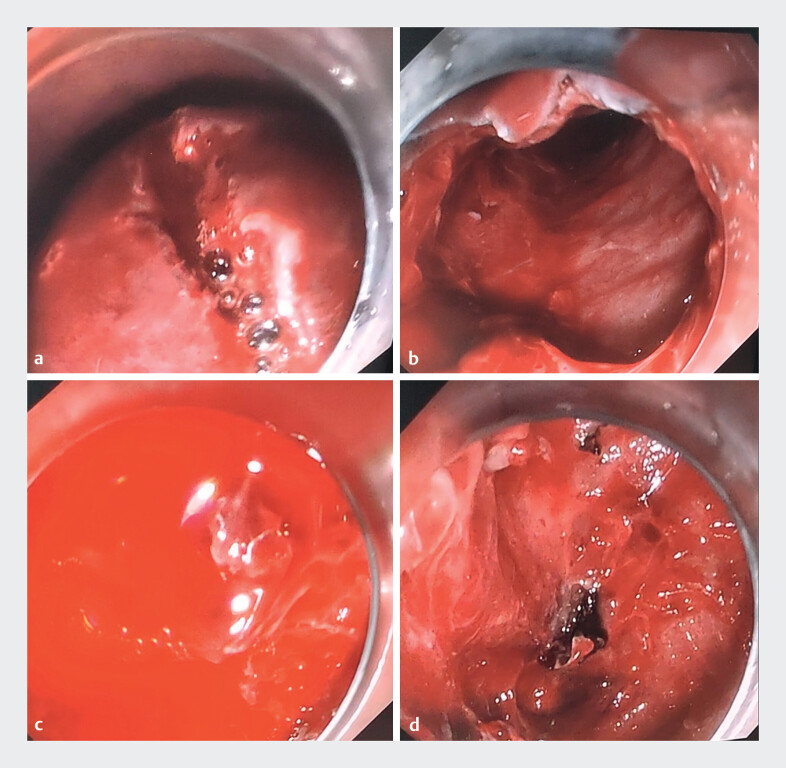
Endoscopic images showing: **a** the re-incised swollen tunnel, containing a large number of dark red blood clots; **b** the tunnel after clearance of all the blood clots; **c** active oozing of blood from a blood vessel in the submucosa of the cardia; **d** the appearance after hemostasis was successfully achieved by electrocoagulation.

Clearance of clots from the tunnel and identification of the bleeding site allows successful management of delayed bleeding after peroral endoscopic myotomy for achalasia.Video 1

Re-examination 10 days after the operation showed no evidence of further bleeding in the tunnel ( Fig. 2 ), and the patient was then discharged from the hospital.

**Fig. 2:**
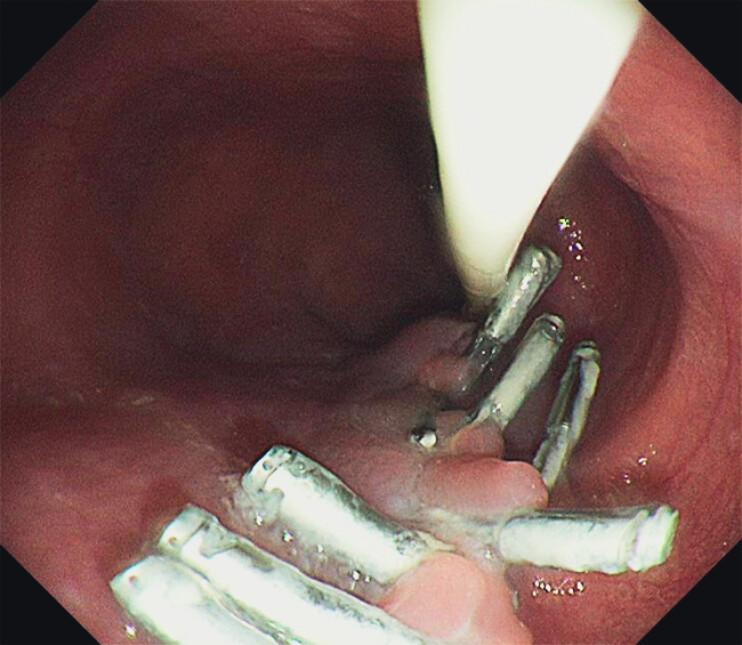
Endoscopic image showing no evidence of further bleeding in the tunnel and the clips still in position to seal the tunnel opening.

Bleeding within the tunnel after a POEM operation is extremely rare 1 . Once it occurs, dealing with it can be troublesome 2 . In cases of massive bleeding, the key to hemostasis lies in clearing the blood clots inside the tunnel to allow identification of the bleeding site.

Endoscopy_UCTN_Code_CPL_1AH_2AZ_3AZ

## Notes

Endoscopy E-Videos https://eref.thieme.de/e-videos
*E-Videos* is an open access online section of the journal *Endoscopy* , reporting on interesting cases and new techniques in gastroenterological endoscopy. All papers include a high-quality video and are published with a Creative Commons CC-BY license. Endoscopy E-Videos qualify for HINARI discounts and waivers and eligibility is automatically checked during the submission process. We grant 100% waivers to articles whose corresponding authors are based in Group A countries and 50% waivers to those who are based in Group B countries as classified by Research4Life (see: https://www.research4life.org/access/eligibility/ ). This section has its own submission website at https://mc.manuscriptcentral.com/e-videos .
